# Poly(vinyl alcohol)/gelatin Hydrogels Cultured with HepG2 Cells as a 3D Model of Hepatocellular Carcinoma: A Morphological Study

**DOI:** 10.3390/jfb6010016

**Published:** 2015-01-13

**Authors:** Stefania Moscato, Francesca Ronca, Daniela Campani, Serena Danti

**Affiliations:** 1 Department of Clinical and Experimental Medicine, University of Pisa, via Savi 10, 56126 Pisa, Italy; 2 Department of Surgical, Medical, Molecular Pathology and Emergency Medicine, University of Pisa, via Savi 10, 56126 Pisa, Italy; E-Mails: francesca.ronca@med.unipi.it (F.R.); dcampani@med.unipi.it (D.C.); 3 Laboratory of Creative Engineering & Design, the Biorobotics Institute, Scuola Superiore Sant’Anna, viale R. Piaggio 34, 56025 Pontedera (PI), Italy

**Keywords:** three-dimensional (3D), cancer model, poly(vinyl alcohol) (PVA), gelatin, HepG2, hepatocellular carcinoma (HCC), histology, cell morphology

## Abstract

It has been demonstrated that three-dimensional (3D) cell culture models represent fundamental tools for the comprehension of cellular phenomena both for normal and cancerous tissues. Indeed, the microenvironment affects the cellular behavior as well as the response to drugs. In this study, we performed a morphological analysis on a hepatocarcinoma cell line, HepG2, grown for 24 days inside a bioartificial hydrogel composed of poly(vinyl alcohol) (PVA) and gelatin (G) to model a hepatocellular carcinoma (HCC) in 3D. Morphological features of PVA/G hydrogels were investigated, resulting to mimic the trabecular structure of liver parenchyma. A histologic analysis comparing the 3D models with HepG2 cell monolayers and tumor specimens was performed. In the 3D setting, HepG2 cells were viable and formed large cellular aggregates showing different morphotypes with zonal distribution. Furthermore, β-actin and α5β1 integrin revealed a morphotype-related expression; in particular, the frontline cells were characterized by a strong immunopositivity on a side border of their membrane, thus suggesting the formation of lamellipodia-like structures apt for migration. Based on these results, we propose PVA/G hydrogels as valuable substrates to develop a long term 3D HCC model that can be used to investigate important aspects of tumor biology related to migration phenomena.

## 1. Introduction

Hepatocellular carcinoma (HCC) accounts for 70%–80% of the total liver cancers and many efforts have been made in the latest decades to find new and more efficient therapies to prolong the overall survival of patients [1,2]. Systemic chemotherapy after surgery, adjuvant therapies and new molecular targeted drugs have guaranteed only a partial success due to their limited case application. Moreover, as in other cancer treatments, also in HCC drug resistance represents a major challenge [3,4]. Liver transplantation is considered the last therapeutic option owing to the low number of donors, the use of immunosuppressive drugs, which can cause severe collateral effects, and the high frequency of both viral and bacterial infections [3,4]. It appears thus evident that innovative therapies, more effective, selective and possibly patient-specific, are greatly expected.

*In vitro* models have been developed to allow the study of tumor cell behavior in response to therapies [5]. The bidimensional (2D) cultures have made it possible to investigate different cellular phenomena such as proliferation, differentiation or function. Anyway, in tissues or organs, cells are naturally organized in three-dimensional (3D) structures interacting with both other cell types and the surrounding extracellular matrix (ECM). As a consequence, inherent limits of 2D models, such as cell phenotype and ECM dissimilarities with respect to those of the tumor and insufficient microenvironment complexity, have revealed to affect the reliability of these models [6,7]. To overcome such difficulties, new 3D *in vitro* models have been recently proposed [8,9,10]. Despite that, so far the pharmacological studies to evaluate the cytotoxicity or the pharmacokinetic of new drug compounds have been mainly performed on 2D models.

In normal (*i.e.*, non-cancerous) cell cultures, phenotype and genotype differences have been documented between 2D and 3D *in vitro* models, so as many other biological phenomena including cell proliferation, differentiation or function [11]. Some authors have highlighted that these differences are even more profound when cancer cells are cultured in a 3D structure compared to a monolayer [12,13]. These findings rendered it appealing the consolidation of the tissue engineering approaches to understand tumor cell dynamics involved in proliferation, metastasis and pharmacological response [7,10,14].

Indeed, in cancer applications, tissue engineering offers innovative options focusing on the study of new biomimetic and biocompatible materials that can support the proliferation and the viability of cells to create not only normal, but also pathological 3D tissue models [15,16]. The development of advanced 3D *in vitro* models can disclose the comprehension of tumor progression and behavior in different experimental conditions. One of the key factors that influence successful outcomes in the field of tissue engineering is the identification of an appropriate scaffold that resembles the selected *in vivo* environment.

Some tissue engineering approaches have been used by researchers to create bio-hybrid constructs as a 3D model of HCC. Scaffolds both of synthetic and biologic origins have been investigated for the growth and function of HepG2 cells, the latter considered as a consolidated cellular model of HCC. The synthetic matrices tested for 3D culture of HepG2 included beads of apatite or silica, porous polymeric scaffolds based on poly(vinyl alcohol) (PVA), tetraethoxysilane/polydimethylsiloxane, polyurethane, and polystyrene [13,17]. In these scaffolds, the HepG2 cells generally revealed an enhanced metabolic activity compared to the 2D conditions. Moreover, these findings suggested that the 3D environment strongly influenced drug efficacy, thus resembling more closely the tumor phenomena occurring *in vivo* [13]. Among the biological materials, proteins and polysaccharides have been used to prepare scaffolds. Matrigel^®^, collagen and self-assembled peptide nanofibers have been reported in some studies, showing both enhanced hepatic function and promotion of malignant phenotype in HepG2 cells, with respect to 2D cultures [18,19]. Finally, 3D HCC models have been fabricated via HepG2 encapsulation inside alginate hydrogels [20]. In these culture conditions, the cells were not able to proliferate, even if they were found to be viable and functionally active. This phenomenon was supposed to be a consequence of ineffective cell adhesion to the biomaterial matrix.

Hydrogels represent an intriguing class of biomaterials for scaffold fabrication, owing to the high water content that they can retain, the similarity with the ECM and their moldability into plenty of different shapes [21,22,23]. In particular, PVA is a synthetic polymer that can form hydrogels under a physical cross-linking, obtained by repeated freeze-thawing cycles of aqueous polymeric solutions [24]. Bioartificial hydrogels, combining PVA with different biologic molecules, such as dextran, hyaluronic acid and gelatin (G), have been proposed for tissue engineering applications [25]. Such hydrogels for certain weight composition ratios between the biological and synthetic components displayed anisotropic lamellar structures and small size macropores that could represent interesting features to mimic the liver structures.

This study was aimed at developing a novel 3D HCC model to enable the comprehension of biological processes involved in tumor cell migration, focusing on the morphological aspects of cancer cells. We prepared PVA/G hydrogels with weight composition 80/20 under sterile conditions. The morphology of PVA/G scaffolds was analyzed via scanning electron microscopy (SEM) and mercury intrusion porosimetry. These hydrogels were seeded with HepG2 cells and cultured *in vitro* up to 24 days. Viability of cell/scaffold constructs was monitored along the culture time using a metabolic activity assay, and double-checked at the endpoint through the expression of hypoxanthine-guanine phosphoribosyltransferase (HPRT), as a fundamental metabolic enzyme, via western blotting. The morphology of cell/scaffold constructs was analyzed via histology and compared to those of HepG2 cells in 2D cultures and HCC specimens. Finally, the expression of β-actin, investigated via immunohistochemistry and western blotting, and the immunolocalization of α5β1 integrin were evaluated and put in relation to HepG2 cell morphology.

The establishment of an HCC model mimicking cancer cell migration and invasion is expected to give new insight on the tumor biology, thus paving the way to the research and development of more effective therapies.

## 2. Results

### 2.1. Morphological Characterization of PVA/G Hydrogels

**Figure 1:**
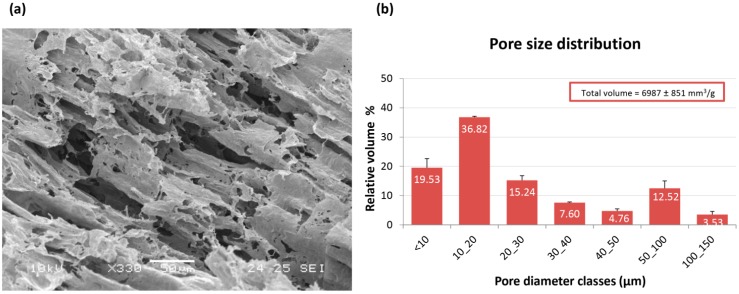
Morphological analysis of PVA/G scaffolds: (**a**) SEM micrograph showing the inner structure of the scaffold; (**b**) bar graph showing the porosimetric analysis of the scaffold, expressed as relative volume percent for selected poral classes. Data are reported as mean ± SD.

Macroporous hydrogels were prepared under sterile conditions blending water solutions of PVA and G with culture medium (CM) and using freeze-thawing cycles to obtain the physical crosslinking of PVA. The hydrogels assayed in this study had a final PVA/G ratio of 80/20 (w/w), a 93.5% weight water content and a 425% volume increase. SEM analysis of PVA/G hydrogels showed a porous 3D structure arranged as thin directional lamellar structures (Figure 1a). Size and distribution of macropores were investigated via mercury intrusion porosimetry. Data were reported as the percentage of volume occupied by pores whose diameter ranged in selected classes (Figure 1b). The pores with diameters ranging in 10–20 μm seized the biggest volume fraction (36.82% ± 3.12%). On the whole, 71.59% ± 5.03% of the measured volume was due to pores with diameters lower than 30 μm (namely, ranging in 0.007–30 μm), while only 28.41% ± 4.57% of the measured volume derived from pores with larger diameters (namely, ranging in 30–150 μm).

### 2.2. Viability of Cell/Scaffold Constructs

**Figure 2:**
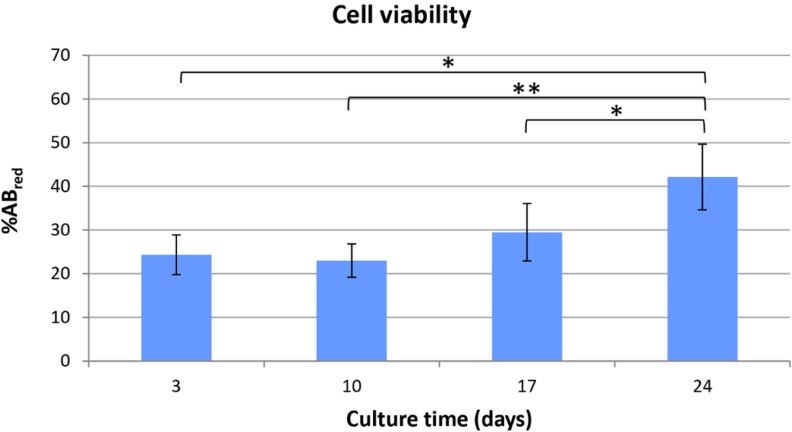
Bar graph reporting the results of alamarBlue^®^ assay at different time-points. Data are reported as mean ± SD; asterisks indicate the following magnitude orders of *p* values: * 10^−3^ and ** 10^−4^.

The alamarBlue^®^ assay was used to monitor the metabolic activity of HepG2 cells along the culture time as a proof of their viability within the scaffolds (Figure 2). The bioassay revealed that the cells were viable up to 24 days in culture inside PVA/G hydrogels.

The metabolic activity, expressed as dye reduction percent (%AB_red_), measured in cell/scaffold constructs on day 3, 10, 17 and 24 after seeding, was 24.35% ± 4.54%, 22.99% ± 3.82%, 29.48% ± 6.58% and 42.12% ± 7.53%, respectively. At the endpoint, the metabolic activity of the constructs resulted significantly higher than that detected at all the previous time-points, showing *p* values equal to 0.0019, 0.0005 and 0.0013 in comparison against day 17, 10 and 3, respectively. No statistically significant differences were revealed when the day 3, 10, 17 time-points were compared among each other.

### 2.3. Histologic Analysis

**Figure 3:**
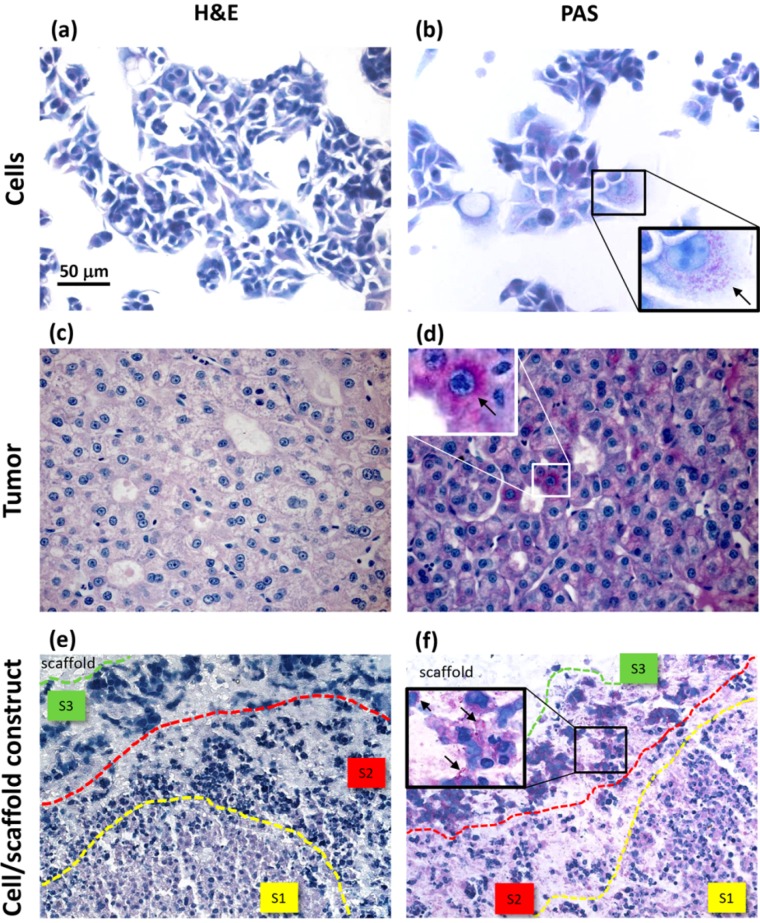
Histochemical analysis of HepG2 cells cultured in monolayers (**a**,**b**); samples of HCC (**c**,**d**) and HepG2 cells cultured inside PVA/G hydrogels (**e**,**f**). For each sample type, H&E (a,c,e) and PAS reaction (b,d,f) are shown. Arrows in (b,d,f) indicate evidence of PAS positivity. S1, S2 and S3 in (e,f) define the areas of different morphotype localization in cell/scaffold constructs.

Morphological analysis of cellular samples were performed using hematoxylin and eosin (H&E) staining (Figure 3a,c,e), while Periodic Acid Shiff (PAS) reaction was used to highlight the presence of glycoproteins and glycogen, as a product of metabolically active hepatic cells (Figure 3b,d,f). Glycoproteins are a class of adhesive proteins that own binding sites enabling cell attachment to the ECM. Specifically, histologic analyses were performed on HepG2 cells, samples of HCC and HepG2 cells cultured inside PVA/G hydrogels. In 2D cultures, HepG2 cells showed a typical stellate shape when distributed at a low density, while a round shape when assembled in cluster-like formations (Figure 3a). In both morphotypes, PAS reaction revealed a weak positivity as only a few cytoplasmatic granules were stained (Figure 3b). The PAS positivity was also observed on tumor sections, in which most cells displayed diffuse staining, while only a reduced number of cells were strongly positive (Figure 3d).

Both PAS reaction and H&E staining showed that hepatocytes reorganized into duct-like formations. Such a differentiative parameter is a hallmark of the HCC tumor (Figure 3c,d). In cell/scaffold constructs, H&E highlighted that HepG2 sprout out inside the hydrogels forming large cellular aggregates within the polymeric matrix. These cellular structures were constituted by different morphotypes according to their localization and three main areas could be recognized (Figure 3e,f). In the central area, indicated as S1, small cells with a big nucleus and an eosinophil cytoplasm were present and arranged to form trabecular-like structures. The peripheral area, indicated as S3, was populated mainly by large cells, strongly basophilic with a flat shape, suggesting a stellate-like morphology.

Furthermore, in this area, PAS reaction was strongly positive both at intra- and extra-cellular level, thus denoting an active secretion of glycoproteins. Between S1 and S3, an intermediate area could be observed, indicated as S2, in which cells displayed pyknotic nuclei and damaged membranes, as a probable result of cellular stress.

**Figure 4:**
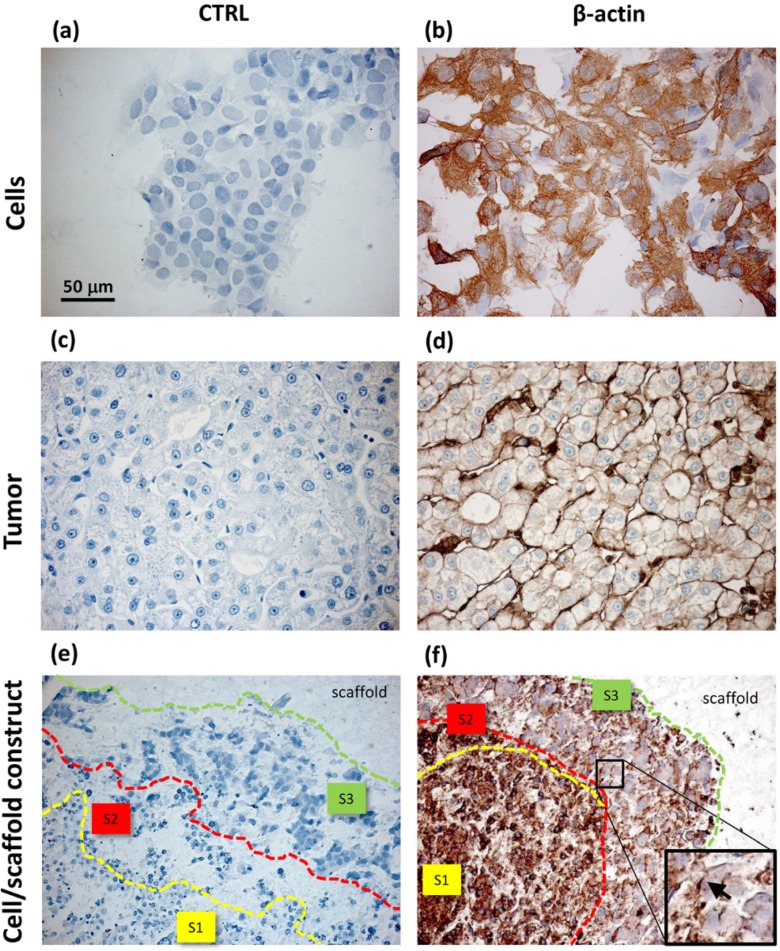
Immunohistochemical analysis of HepG2 cells cultured in monolayers (**a**,**b**); samples of HCC (**c**,**d**) and HepG2 cells cultured inside PVA/G hydrogels (**e**,**f**). For each sample type, negative controls (a,c,e) and β-actin expression (b,d,f) are shown. S1, S2 and S3 in (e,f) define the areas of different morphotype localization within the cell/scaffold constructs. The insert in (f) shows a few cells with a lamellipodial-like expression of β-actin, indicated with an arrow.

In this study, two fundamental proteins related to cell migration were evaluated, namely β-actin and α5β1 integrin. β-actin, a protein involved in cytoskeleton arrangement and remodeling, was investigated via immunohistochemistry and compared to negative controls (CTRL) performed omitting the primary antibody (Figure 4).

HepG2 cells cultured in monolayers showed a high immunopositivity for β-actin with a filamentous pattern distribution (Figure 4b). Differently, in tumor sections, β-actin was preferentially localized under the plasma membrane and in the correspondence of duct lumen, in which cell microvilli protruded (Figure 4d). This immunohistochemical observation was corroborated by the presence of zonal cell distribution in the cell/scaffold constructs, highlighting the presence of different cellular morphotypes identifiable via histochemical staining (Figure 4e,f). The small cells in the central area (S1) were more immunopositive to β-actin than the large cells located in the peripheral area. However, the stellate cells showed a strong and specific immunoreaction localized on a side edge of their membrane (insert in Figure 4f).

**Figure 5:**
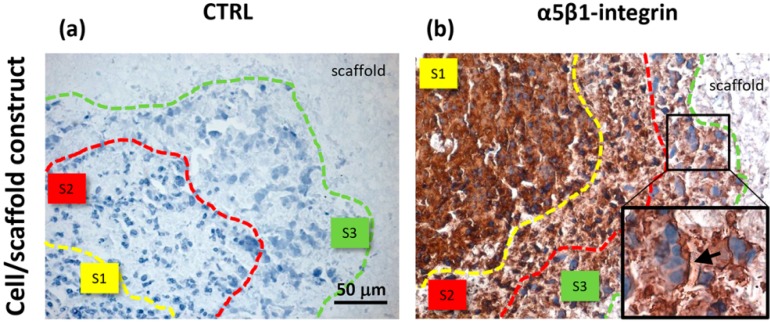
Immunohistochemical analysis of HepG2 cells cultured inside PVA/G hydrogels, showing negative control and α5β1 integrin expression (**a**,**b**). S1, S2 and S3 in (a,b) define the areas of different morphotype localization within the cell/scaffold constructs. The insert in (b) shows a few cells with a side border expression of α5β1, indicated with an arrow.

Immunohistochemistry revealed a similar expression pattern for α5β1 integrin, a cell-matrix transmembrane receptor binding ECM and proteinases expressed by HepG2 cells. The results, showing specific immunopositivity of α5β1 integrin on the side border of cells lying in the S3 zone, are displayed in Figure 5. The anti-integrin α5 antibody here used recognized the intracellular (*i.e.*, cytoplasmatic) domain of the protein, thus highlighting morphological features of the cells.

### 2.4. Western Blotting

**Figure 6:**
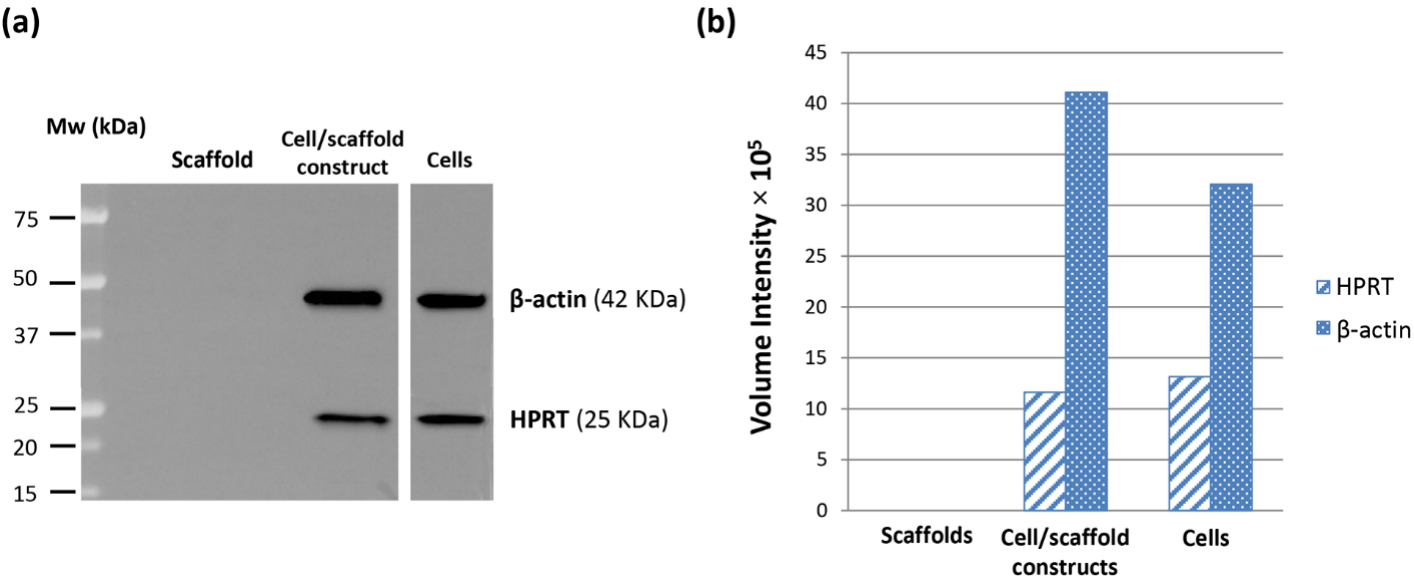
Western blot results of β-actin and HPRT expression in HepG2 cells cultured in monolayers for 4 days (80% confluence) and inside PVA/G hydrogels for 24 days. Hydrogels without cells were tested as negative controls. The immunoblot reaction is shown in (**a**) and the band analysis in (**b**).

Immunoblot assay was performed on HepG2 cells cultured both in monolayers (2D) and inside PVA/G hydrogels (Figure 6). Results showed a high expression of β-actin in cell/scaffold constructs, thus confirming the previous immunohistochemical data. Moreover, the volume intensity evaluation of the bands revealed that in the cell/scaffold constructs β-actin was expressed at a higher concentration than in HepG2 monolayer cultures, taking in account that the same total protein amount was loaded and separated by the SDS-PAGE gels. Its calculated values for band volume intensity of cell/scaffold constructs and monolayer cells were 41.10 × 10^5^ and 32.06 × 10^5^, respectively (Figure 6b).

HPRT was evaluated via immunoblotting as a marker of metabolic activity (Figure 6). The band volume intensities showed a reading of 11.61 × 10^5^ and 13.16 × 10^5^, for cell/scaffold constructs cultured for 24 days and monolayer cells cultured for 4 days (up to 80% confluence), respectively (Figure 6b). No appreciable difference in the HPRT expression was revealed between the two culture conditions.

## 3. Discussion

In this report, we propose a new 3D *in vitro* model of HCC based on HepG2 liver cells seeded inside PVA/G hydrogels and cultured for long term (namely, 24 days) to provide a platform that will potentially enable the study of biological processes driven by the migratory capability of cancer cells. We performed a morphological investigation as a preliminary study to reveal migration-related changes of cell morphology inside a 3D environment. Our results demonstrated that HepG2 cells modified their morphological features and cytoskeletal organization according to their zonal localization within the cellular aggregates formed inside the scaffolds. These findings highlighted the importance of the 3D microenvironment for the comprehension of the mechanisms occurring in tumor progression.

Some authors have reported difficulties related to the use of hydrogels as scaffolds, owing to some practical issues, such as gel preparation, storage and variability [13]. Differently, PVA/G hydrogels are easy to prepare [24,25]. Additionally, they can be produced as sterile substrates, since the polymer is dissolved by autoclaving and the gelatin aqueous solution at the used concentration can be sterile-filtered. Moreover, the freeze-thawing procedure can be performed using sealed plates in order to preserve the material sterility. These bioartificial hydrogels combined the structural properties of the synthetic polymer (PVA) with the high cell affinity of the protein (G), thus enhancing the scaffold cytocompatibility [25]. Moreover, it has been documented that blending PVA with G up to 20% (w) to form hydrogels gave rise to directional lamellar structures [25]. To dilute the initial PVA/G mixture up to the desired final PVA concentration, complete CM was added during preparation. In this way, the hydrogels obtained were ready to use as scaffolds, since they already incorporated cell culture supplements and provided cell suitable osmolarity in the liquid phase. The proposed method for hydrogel preparation can be easily afforded by biological laboratories that are not equipped with scaffold fabrication facilities, thus representing a user-friendly procedure to prepare substrates for 3D cancer models.

From a morphological point of view, PVA/G scaffolds appeared to be as macroporous substrates with anisotropic lamellar structures outlined by thin mesoporous polymeric surfaces. Such a scaffold morphology reminded the trabecular structure of liver parenchyma and appeared suitable for liver cell disposal. In the dried hydrogels, about ¾ of the empty volume derived from pores with diameters lower than 30 µm, while greater pore diameter classes were present at a lower extent. These results were in line with previous observations [25]. It has been documented that the poral features of the scaffolds play a key role in cell colonization, morphology and function [26]. Moreover, the scaffold inner architecture, as a result of spongy or nanofiber-interspace pores, can be a discriminant in cancer modeling [17,27]. Plain PVA porous scaffolds, as substrates for HepG2 cells, have been firstly proposed by Kataoka *et al.* [17]. In this study, commercial PVA scaffolds with 130 µm and 200 µm were investigated and compared with other polymeric scaffolds with similar porosity. The authors reported a reduced cell proliferation and colonization that were attributed to the surface properties of PVA, supposed to impair an efficient cell clustering. The presence of biological macromolecules in PVA hydrogels has been reported to modify their internal architecture and possibly their surface properties [25]. As an example, PVA/G in the form of sponges, has been documented to act as a valuable support for fibroblast growth and ECM synthesis [28]. In our experimental conditions, the use of bioartificial PVA hydrogels with pore size lower than that described by Kataoka *et al.* resulted well suitable for HepG2 colonization. However, it has to be outlined that pore size distribution, as conventionally measured on dried samples, can be only representative of materials, such as hydrogels, that meet the target of swelling. After preparation, PVA/G hydrogels appeared as chewy gels with a strong capacity of liquid retention as demonstrated by a 93.5% (w) weight water content and a 425% volume increase. In the first instance, this aspect challenged the cell seeding events. However, Hamilton syringes made it possible to perform cell seeding via inoculation with an acceptably low variability, which was confirmed by the outcomes of alamarBlue^®^ assay. Indeed, the HepG2 cells seeded inside PVA/G hydrogels were able to maintain their metabolic activity for more than 2 weeks and showed a statistically significant increase at the endpoint. At that time, the occurrence of neither apoptotic nor highly relevant necrotic phenomena could be documented (data not shown). Therefore, it cannot be excluded that the cells underwent a lag stage followed by an intense proliferation phase. The metabolic activity outcome was corroborated by the expression of HPRT, revealed via western blotting. HPRT is a transferase that catalyzes the conversion of hypoxanthine to inosine monophosphate, and guanine to guanosine monophosphate. This enzyme plays an important role in the generation of purine nucleotides throughout the purine salvage pathway. The presence of HPRT in the constructs confirmed active cell metabolism after 24 days in culture.

Other studies have evaluated several devices, fabricated using synthetic or biological materials for HepG2 3D culture, also in dynamic conditions [13,17,18,19,20]. However, these investigations were performed at earlier culture times, *i.e.*, up to 14 days. Moreover, an extensive morphological analysis of cell/scaffold constructs has not been reported so far. Indeed, Bokhari *et al.* have shown morphological features of HepG2 cells in polystyrene scaffolds via electron microscopy [13]. Similarly, Kataoka *et al.* have reported cell presence in the constructs via SEM and basic histologic analysis (H&E) [17]. In our opinion, histology represents a valuable tool to highlight important phenomena, such as tumor cell migration, that can be more efficiently observed in 3D models at later culture times.

Many authors have referred to spheroids as 3D cancer models [29]. However, these structures have been documented to mimic only some aspects of the tumor microenvironment [7,30]. Tissue-engineered constructs have been recently proposed as more reliable cancer models than spheroids [7,10,16,30]. Indeed, if cultured inside biomaterial scaffolds, tumor cells are more likely to reproduce the *in vivo* tissue architecture and be viable for extended periods [13,27]. In our investigation, we performed a histologic analysis to detect cell morphology and the expression of biosynthesis, structural and interaction molecules, such as glycoproteins, β-actin and α5β1 integrin. In the cell/scaffold constructs, cells were found to be arranged in large aggregates, either located at the inoculation spots or in separate sites of the hydrogel, that were all characterized by three different areas, in which diverse HepG2 morphotypes were present. In the central area, small cells were arranged to form trabecular-like structures, similar to the liver parenchyma, while the peripheral area was populated by large stellate cells with an active secretion of glycoproteins. In between these two areas, stressed cells and a few necrotic phenomena were observed. The presence of a necrotic zone has been detected also in tumor spheroids, being preferentially localized in the core as a critical area of metabolite distribution [31]. Differently, our constructs showed distinct cellular agglomerates, each one characterized by some evidence of necrotic cells only in an intermediate layer between two different cell morphotype areas. This phenomenon thus seemed not to be a consequence of metabolite gradients.

Unlike the cells localized in the most internal layers of the cellular aggregates, which showed the highest and uniformly distributed β-actin and α5β1 integrin expression, the cells in the outer layer displayed a specific β-actin and α5β1 integrin immunopositivity on a side border of their membrane, thus suggesting the formation of lamellipodia-like structures. The regulation and reorganization of cytoskeleton proteins, such as β-actin, has been invoked as a key factor in cancer cell migration, through the formation of lamellipodia and invadopodia/podosomes [32]. Lamellipodia are the main cellular structures devoted to the cell-substrate interactions, enabling the driving force for cell migration induced by chemoattractant gradients [32]. This fact is corroborated by a similar localization of α5β1 integrin in our samples. On the basis of these data, our findings suggested that the stellate cells on the frontline layers of cellular aggregates were interacting with the PVA/G hydrogel surfaces modifying their cytoskeletal filaments and triggering their connections with the outer ECM molecules in order to migrate across the scaffold pores.

Comparing to HepG2 monolayers, cell/scaffold constructs represented an advanced model of HCC. Indeed, in 2D cultures, the territorial difference between the two morphotypes was not highly marked and no cytoskeletal reorganization was observed. The comparison of the 3D model with HCC specimens appeared more difficult, since the tumor tissues analyzed in this study showed well defined and mature characteristics typical of a late differentiation stage. In these tumor sections it was not possible to observe dynamic phenomena, such as cell migration.

All in all, PVA/G hydrogels appeared to promote the long-term growth and the differentiation of the hepatocarcinoma cell line HepG2, allowing the formation of more complex 3D structures that can be useful to study the biology of HCC tumors. In particular, these 3D constructs could be tested for drug susceptibility and possibly give new insight to target, develop or refine cancer therapies. These bioartificial scaffolds are thus proposed as a valuable tool to develop a long term 3D model of HCC.

## 4. Experimental Section

### 4.1. Preparation of PVA/G Hydrogels

PVA/G hydrogels were prepared modifying a procedure described in a previous study [25] in order to obtain sterile scaffolds ready to use. Briefly, a 10% (w/v) solution of PVA (99% hydrolyzed, *M*_w_ 85,000–146,000; Sigma-Aldrich, Milan, Italy) in distilled water was prepared using an autoclave cycle, set at 120 °C and 20 min. The PVA solution was cooled down to 60 °C under magnetic stirring, opened inside a cell culture flow cabinet and blended with a 3% (w/v) sterile filtered solution of gelatin (G; from skin bovine type B, 75 Bloom; Sigma-Aldrich) in distilled water to obtain a bioartificial mixture with a PVA/G ratio of 80/20 (w/w). This mixture was complemented with complete CM, consisting of red phenol-free Dulbecco’s modified Eagle Medium (D-MEM) supplemented with 10% fetal calf serum, 1% non-essential aminoacids, 200 mM L-glutamine and 1% penicillin-streptomycin (all from Sigma Aldrich) to reach a final PVA concentration of 2.5% (w/v). The PVA/G blend in CM was loaded inside 96-well culture plates, at 200 µL/well, using sterile tips. After being filled with the solution, the well-plates were tightly closed to preserve the sterility and processed to obtain hydrogels via 8 freeze-thawing cycles. The first cycle consisted of overnight freezing at −20 °C followed by 30 min defrosting at room temperature. In the subsequent cycles −20 °C freezing was applied for 1 h. At the end, the well-plate was opened under the cell culture flow cabinet and excess supernatant was removed. The gravimetric water content percent and the volume increase percent were calculated by weight and size measurements, with respect to wet and dry condition, respectively.

### 4.2. Morphological Analysis of the PVA/G Hydrogels

The specimens underwent preliminary dehydration through a graded ethanol/water solutions up to anhydrous ethanol that was removed using the Critical Point method (Balzers CPD030, Oerlikon Balzers, Balzers, Liechtenstein) to obtain dried hydrogels. The inner structure of the hydrogels was analyzed via SEM using a Jeol JSM-5600 LV (Jeol, Tokyo, Japan). The samples were mounted on aluminum stubs and coated with gold (Edwards Sputter Coater S150B, Edwards, NY, USA) prior to examination via SEM under an accelerating voltage of 10–15 kV.

Poral features of the hydrogels were investigated via mercury intrusion porosimetry (*n* = 3). Pore size ranging in 0.007–120 μm diameters was evaluated by Hg intrusion using a porosimeter (Pascal 140, Carlo Erba, Pomezia, Italy) equipped with an automatic recording of intruded Hg volume. The pore volume distribution was obtained from the derivative curve of the cumulative intruded pore volume as function of pore diameter. This latter parameter is related to the measured pressure according to the Washburn’s model equation developed for cylindrical shape pores [33]:

(1) $$d=10\cdot 4\gamma \cos\frac{\theta }{P}$$

Stating that the cylindrical diameter *d* (μm) of the Hg-filled pores is inversely proportional to the intrusion pressure *P* (kg/cm^2^), when the surface tension of Hg γ and the contact angle θ between Hg and the material are constant (γ = 0.48 N/m). Contact angles of 141.3° were measured for these hydrogels.

### 4.3. Cell Cultures

The human hepatocarcinoma cell line HepG2 (ECACC, Sigma-Aldrich) was used to prepare cell/scaffold constructs. HepG2 cells were expanded using complete CM and passaged every 4–5 days using 1% Trypsin/EDTA solution (Sigma-Aldrich) for detachment. For 2D studies, HepG2 were seeded on glass slides as multiple spots of 1 × 10^4^ cells each, and let adhere for 2 h, covered with complete CM and maintained in culture until the confluence was reached. Sterile PVA/G hydrogels were placed into 24-well plates and HepG2 cells were seeded into the scaffolds using a Hamilton syringe (Hamilton Bonaduz, Bonaduz, Switzerland) equipped with a thin needle (gauge 19).

Specifically, 1 × 10^6^ cells were suspended in 20 μL of complete CM and inoculated into the scaffold through multiple shots. Cells were let adhere for 2 h before adding 1 mL of complete CM inside the wells. All the cell cultures were conducted in a humidified incubator under standard conditions, namely, 37 °C, 95% humidity and 5%/95% CO_2_/air mixture.

### 4.4. Viability of Cell/Scaffold Constructs

Viability of HepG2 cells cultured inside PVA/gel hydrogels was evaluated using alamarBlue^®^ assay (Life technologies, Carlsbad, CA, USA). Metabolically active cells are able to reduce the bioassay indicator resulting in color change of the CM. Moreover, samples can be assayed at different culture time-points thanks to the negligible toxicity of the dye. Data were acquired according to manufacturer instructions, and were expressed as percentage of reduced alamarBlue^®^ (%AB_red_). Briefly, samples (*n* = 6) and controls, *i.e.*, scaffolds with no cells (*n* = 6), were incubated for 3 h at 37 °C with the alamarBlue^®^ dye diluted in CM according to the manufacturer’s recommendations. Viability tests were performed every 7 days after seeding. At each time-point, 100 μL of supernatant from sample or control was loaded in 96-well plates; then, excess supernatant was removed from the cultures and replaced with fresh CM. The absorbance (λ) of supernatants was measured with a spectrophotometer (Microplate Reader Model 680, Biorad, Hercules, CA, USA) under a double wavelength reading (570 nm and 600 nm).

Finally, %AB_red_ was calculated correlating the absorbance values and the molar extinction coefficients of the dye at the selected wavelengths, following the protocol provided by the manufacturer. The equation applied is shown below:

(2) $$\%{\text{AB}}_{\text{red}}=100\times \frac{\left(117,216\cdot \lambda_{\text{sample}@570\mathrm{nm}}-80,586\cdot \lambda_{\text{sample}@600\mathrm{nm}}\right)}{\left(155,677\cdot \lambda_{\text{control}@600\mathrm{nm}}-14,652\cdot \lambda_{\text{control}@570\mathrm{nm}}\right)}$$

### 4.5. Tumor Specimens

Tumor specimens of three patients affected by HCC with no evidences of cirrhosis or fibrosis could be accessed in the framework of a retrospective study on hepatic tumors conducted by the Anatomic Pathology Unit at the Hospital of Cisanello, Pisa, Italy. Sections, 3-μm thick, were collected and treated for histochemistry and immunohistochemistry.

### 4.6. Histologic Processing of Cell/Scaffold Constructs

At the end of the culture, samples were treated for histologic analysis. Briefly, both cell/scaffolds constructs and plain scaffolds were fixed in 4% neutral buffered formalin (0.1 M, pH 7.2) at 4 °C overnight, washed in 1× phosphate saline buffer (PBS; Sigma-Aldrich) four times for 15 min and finally rinsed in 70% ethanol until processing. Thereafter, the samples were dehydrated through a series of graded ethanol: 80% and 90% ethanol for 30 min, 95% ethanol for 60 min and absolute ethanol for 6 h. Subsequently, the samples were clarified in xylene twice for 2 h, rinsed in liquid paraffin at 60 °C for 4 h and wax embedded. Specimen sections 5-μm thick were obtained, mounted onto slides and stored until histologic processing.

### 4.7. H&E Staining

The sections were deparaffinized by immersion in xylene twice for 7 min and in absolute ethanol three times for 7 min, stained with hematoxylin (Sigma-Aldrich) for 5 min, washed in tap water for 5 min, counterstained with eosin (Sigma-Aldrich) for 1 min, then washed in tap water and dehydrated in 95% ethanol for 5 min and in absolute ethanol three times for 7 min. After clarification in xylene three times for 7 min, the specimens were mounted with DPX mountant medium (Fluka, Buchs, Switzerland).

### 4.8. PAS Reaction

After deparaffination and washing in dd-H_2_O, the samples were incubated in 0.1% periodic acid in double-distilled H_2_O (Fluka) for 10 min, air dried and treated with Schiff reagent (Sigma-Aldrich) for 15 min. After washing in tap water for 10 min, the sections were counterstained with hematoxylin for 5 min, washed in tap water for 5 min, dehydrated and mounted in DPX medium.

### 4.9. Immunohistochemistry

Specimen sections were deparaffinized and brought up to water; samples permeabilization was performed using 0.2% Triton X-100 (Sigma-Aldrich) in 1× PBS for 10 min. Quenching of peroxidase was reached by incubation in 0.6% H_2_O_2_ in methanol in the dark for 15 min. In order to block the aspecific binding sites, the samples were incubated with goat serum (Vektor Lab, Burlingame, CA, USA) diluted 1:20 in 1× PBS at 37 °C for 20 min. After washing, the samples were incubated with anti β-actin mouse monoclonal antibody (A-5316, Sigma-Aldrich) and anti-integrin α5 antibody (AB1928, Millipore, Merck KGaA, Darmstadt, Germany) diluted 1:2000 and 1:100, respectively, in 0.1% BSA/1× PBS in moist chamber at 4 °C overnight. Negative controls were performed incubating some sections with 0.1% BSA/1× PBS only. Next day, the samples were incubated with a goat anti-mouse biotinylated secondary antibody (Vektor Lab) diluted 1:200 in 1.5% goat serum/1× PBS solution for 60 min and then with streptavidin (Vectastain Elite ABC Kit Standard, Vektor Lab) for 30 min. In order to reveal the reaction, the sections were incubated in the chromogenic substrate solution at 0.5 mg/mL 3,3'-diaminobenzidine tetrahydrochloride containing 0.02% H_2_O_2_ (Amresco, Solon, OH, USA) for 5 min in the dark, counterstained with hematoxylin for 30 s and washed in tap water for 1 min. Finally, the sections were dehydrated and mounted as previously described. In the second part of reaction, after each passage, washing in 0.01% Triton/1× PBS was performed. The treated histologic sections were observed with a DMRB Leica microscope (Leica Microsystems, Wetzlar, Germany).

### 4.10. Western Blotting

At the endpoint of the culture, both cell/scaffolds constructs (*n* = 3) and controls (scaffolds without cells as a negative control, and cells grown in monolayer up to 80% confluence—4 days—as a positive control) were washed in D-PBS and resuspended in a cell lysis buffer (50 mM Tris-HCl, pH 7.4, 150 mM NaCl, 2 mM EDTA, 1% NP-40, and antiproteases 1×, all from Sigma Aldrich). Samples were then disrupted using two 30 s pulses by Micro-Ultrasonic Cell Disrupter (Vineland, NJ, USA) set at 50% output in an ice bath. Sample lysates were centrifuged at 15,000 rpm for 20 min at 4 °C and protein concentration in the supernatants was determined by the bicinchoninic acid (BCA; Pierce, Rockford, IL, USA) microplate method. Proteins (30 µg/lane) were separated on a 4%–15% polyacrylamide gel (BioRad, Hercules, CA, USA) under reducing conditions, transferred to a nitrocellulose membrane (Trans Turbo Blot system, BioRad). The membrane was blocked with 4% dry non-fat milk in 0.1% Tween/Tris Buffer Saline (T-TBS) and incubated overnight at 4 °C with anti β-actin mouse monoclonal antibody (A-5316, Sigma-Aldrich) or anti-HPRT rabbit monoclonal antibody (ab109021, Abcam, Cambridge, UK) diluted 1:2000 or 1:10,000 in T-TBS, respectively. Anti-mouse or anti-rabbit Horse Radish Peroxidase (HRP)-conjugated antibodies (KPL, Gaithersburg, MD, USA), both diluted 1:2000 in 4% dry non-fat milk in T-TBS for 1 h at room temperature, were used as secondary antibodies and the immunocomplexes were detected by chemiluminescence (ECL clarity, BioRad) using Chemi-Doc XRS+ (BioRad). The data were analyzed using Image Lab software (BioRad).

### 4.11. Statistical Analysis

Quantitative data were presented as descriptive [mean ± standard deviation (SD)] and inferential statistics (*p* values). Statistical significance was evaluated with two-tailed *t*-test for paired data, followed by Bonferroni correction. Significance was set at *p* < 0.05.

## 5. Conclusions

This study was aimed at developing a new 3D *in vitro* model of HCC usable at late time-points to investigate tumor cell migration within a 3D environment. Specifically, a preliminary morphological investigation was performed to disclose morphological changes occurring in HepG2 cells grown for 24 days inside PVA/G hydrogels. From a morphological point of view, these bioartificial scaffolds showed anisotropic lamellar structures that reminded the trabecular structure of liver parenchyma. The cell/scaffold constructs were compared to HepG2 monolayers and tumor specimens via histology. In the 3D models, HepG2 cells were metabolically active, as demonstrated by glycoprotein synthesis, alamarBlue^®^ assay and HPRT expression. Large cellular aggregates were observed showing different morphotypes with zonal distribution associated to a peculiar β-actin and α5β1 immunolocalization. Specifically, the frontline cells were morphologically different from the inner cells and characterized by strong immunoreactions for both proteins, localized on a side border of their membrane, thus suggesting the formation of lamellipodia-like structures apt for migration. This study proposes PVA/G hydrogels as user-friendly and valuable substrates to develop long term 3D HCC models that can be useful to investigate important aspects of tumor biology related to cell migration and metastasis.
